# Baclofen and catatonia: a case report

**DOI:** 10.11604/pamj.2022.43.198.38403

**Published:** 2022-12-21

**Authors:** Jamir Pitton Rissardo, Sai Nikhil Konduru, Vinay Kumar Gadamidi, Ana Letícia Fornari Caprara

**Affiliations:** 1 Medicine Department, Federal University of Santa Maria, Santa Maria, Brazil,; 2 Medicine Department, American International Medical University, Gros Islet, Saint Lucia,; 3 University of Nebraska Medical Center, Omaha, United States of America

**Keywords:** Baclofen, lioresal, catatonia, psychosis, case report

## Abstract

Baclofen was approved for medical use in the United States in 1977 by Food and Drug Administration. Serious adverse effects associated with this medication are uncommon at usually prescribed doses. Herein, we present a case of baclofen-induced catatonia in a young-adult female with back pain receiving oral baclofen. A 20-year-old female presented to the emergency department with possible seizure-like activity. It was reported that the patient was suffering from acute back pain and was prescribed baclofen three times a day by her general physician one day before her presentation. Upon further discussion, it was known that following an altercation with her family member, she had attempted suicide by consuming 200 mg of baclofen and then developed rapidly progressive symptoms of aphasia, mutism, and decreased oral intake. Laboratory tests, cerebrospinal fluid analysis, and neuroimaging were unremarkable. Electroencephalogram was normal. Bush-Francis Catatonia Rating Scale score was 27. She showed significant improvement following low-dose lorazepam administration. There are four reports in the literature of catatonia secondary to baclofen. The present report is the first to describe the occurrence of catatonia in a previously healthy individual. Analysis of these cases suggests a relationship between a history of psychotic symptoms and catatonia. All the reports were classified as probable by the Naranjo algorithm.

## Introduction

Baclofen was approved for medical use in the United States in 1977 by Food and Drug Administration. Interestingly, baclofen was initially developed for managing epilepsy. But this drug showed no significant effect on the control of seizure activity. During the 1970s, several animal studies demonstrated good efficacy of baclofen for treating muscle spasticity [1].

Baclofen can be used in many conditions, including spastic paralysis, traumatic spinal injury, stroke, and multiple sclerosis. Also, this muscle relaxant has several off-label uses, such as persistent and chronic hiccups. Some available baclofen dosage forms are oral, transdermal, and intrathecal administration through pump infusion. Serious adverse effects associated with this medication are uncommon at usually prescribed doses. However, baclofen overdose was already related to severe neurological side effects like delirium, seizures, and coma [2]. In this context, baclofen was rarely associated with catatonia, potentially life-threatening muscle spasticity characterized by mutism, posturing, rigidity, and withdrawal. Herein, we present a case of baclofen-induced catatonia in a young-adult female with back pain receiving oral baclofen.

## Patient and observation

**Patient information:** a 20-year-old female presented to the emergency department with possible seizure-like activity. She was previously healthy, and her family history was unremarkable for neuropsychiatric conditions.

**Clinical findings:** upon initial evaluation, she was observed to have abnormal posture, facial expression, and stereotypical movements. She was awake but nonverbal with minimal resistance and combativeness.

**Timeline of current episode:** her father reported that she complained of acute back pain the day before the presentation, and her general practitioner prescribed baclofen three times a day. Upon further discussion, it was known that following an altercation with her family member, she had attempted suicide by consuming 200 mg of baclofen and then developed rapidly progressive symptoms of aphasia, mutism, and decreased oral intake. The patient was brought to the emergency department by family members the day after the onset of symptoms.

**Diagnostic assessment:** she was admitted to the hospital and underwent an extensive workup that included a complete blood count, liver function test, metabolic panel, urine analysis/culture, and sexually transmitted serologies, all of which were within normal limits. An electrocardiogram revealed normal sinus rhythm and a QT interval of 350ms. A lumbar puncture was performed, and cerebrospinal fluid analysis was normal. A cranial computed tomography scan was negative. Brain magnetic resonance imaging was unremarkable, without any acute process and signs of encephalitis. Because of her seizure-like activity, an electroencephalogram was performed and revealed normal background for her age without any epileptic activity.

**Diagnosis:** her symptom and negative workup indicated that physical and mental status examinations were associated with catatonia. Bush-Francis Catatonia Rating Scale (CRS) was remarkable for catatonia, and significant symptoms like stupor, mutism, withdrawal, and staring were noted. She was negative for autonomic stability, hyperactivity, echolalia, and impulsivity.

**Therapeutic interventions:** she showed significant improvement following low-dose lorazepam (3mg/day in divided doses orally) administration.

**Follow-up and outcome of interventions:** initially, her motor and stereotyped movements started to improve. On the third day of hospitalization, she was asymptomatic. During the remaining of her hospitalization, she demonstrated no signs of catatonia. She remained nonrigid, well-versed, and eating adequately. At one and three months, she spoke fluently and moved all extremities. No episodes of psychosis, depression, or mania were observed in long-term follow-up.

**Patient perspective:** *“I felt that I was trapped inside my body”*.

**Informed consent:** a written consent form was obtained from the patient for this publication.

## Discussion

Catatonia is a neuropsychiatric syndrome characterized by abnormal movements, behaviors, and withdrawal. A physical and mental assessment of the present individual displayed signs like stupor, mutism, staring, rigidity, withdrawal, and posturing, supporting the diagnosis of catatonia. Catatonia was assessed using Bush-Francis Catatonia Rating Scale (CRS), and her score was 27. Significant improvement in the motor symptoms following the administration of a benzodiazepine can further support the diagnosis of catatonia [3].

Baclofen, also known as 4-Amino-3-(4-chlorophenyl) butanoic acid, is a lipophilic analog of γ-aminobutyric acid (GABA). The baclofen´s mechanism of action is not fully understood. It is believed that this drug is an agonist of the beta subunit of GABA for mono and polysynaptic neurons in the spinal cord and brain. In this way, baclofen probably reduces spasticity by stimulating inhibitory neuronal signals in the post-synaptic neurons and also reduces the release of excitatory neurotransmitters at the presynaptic neurons [1]. Interestingly, intrathecal baclofen is preferred when the patients are unresponsive to oral therapy, mainly in cases of traumatic brain injury and spinal cord lesions. Baclofen´s abrupt discontinuation can cause seizures and hallucinations [4]. This baclofen withdrawal syndrome is important because it supports the hypothesis that baclofen would have a cortical action.

Baclofen-induced catatonia was rarely reported in the literature. It is worth mentioning that the clinical trial for baclofen approval did not record any patient with catatonic symptoms. A literature search was performed in Google Scholar and Medline/PubMed using a set of terms that included baclofen and catatonia (Table 1) [2,4-6]. Analysis of these cases suggests a relationship between a history of psychotic symptoms and catatonia. All the reports were classified as probable by the Naranjo algorithm [7]. Patients with baclofen-induced catatonia usually present with major clinical manifestations of catatonia, such as mutism, stupor, and rigidity. Pauker and Brown highlighted the role of multiple components influencing catatonic symptoms [5]. They suggested a possible interaction among drugs contributed to the development of catatonia.

**Table 1:** literature review of catatonia associated with baclofen

Reference	Country	Age/ sex	Baclofen dose and indication	Concurrent medications	Presentation	Treatment	Note
Pauker *et al*. (1986)	USA	48/F	30 mg/day; persistent lower extremity muscle spasm	Haloperidol, thioridazine, imipramine, levorphanol, hydromorphone	Post-surgery, patient developed delirium, which was treated with antipsychotics. Baclofen was started for muscle spasm. She developed catatonia within three weeks	Catatonia resolved with discontinuation of baclofen. Recurrence of the catatonic symptoms with baclofen rechallenge.	Baclofen-rechallenge revealed
Nahar *et al*. (2017)	India	30/F	300mg orally, one time	Antipsychotics and parenteral sedation	Patient presented with encephalopathy and psychiatry symptoms. She was observed to have catatonic symptoms in the form of posturing, staring, negativism, reduced food intake, ambitendency, and mutism	Catatonia symptoms resolved with lorazepam 3 mg/day	Suicide attempt. CRS 20.
Dcruz *et al*. (2019)	India	63/M	60 mg/day; alcohol use disorder	Disulfram, hypoglycemics, hypolipidemics	Patient was detoxified with diazepam. He was discharged on disulfram and baclofen for alcohol use disorder. The patient developed catatonia after three weeks of baclofen therapy	Catatonia resolved with lorazepam 2 mg/day. Reappearance of catatonia due to continuity of baclofen. Lorazepam was started and baclofen discontinued.	CRS 21. Prior episode of psychosis with catatonic symptoms.
Shaw *et al*. (2022)	USA	19/M	Intrathecal baclofen; muscle spasticity in cerebral palsy	None	Patient developed mutism, staring, withdrawal, and autonomic instability (tachycardia)	Intravenous lorazepam	Misdiagnosed as malfunction of his intrathecal baclofen pump
Rissardo *et al*. (2022)	Brazil	20/F	200mg orally, one time	None	She was observed to have abnormal posture, facial expression, and stereotypical movements. She was awake but nonverbal with minimal resistance and combativeness	Lorazepam 3 mg/day was started	Suicide attempt. CRS score 27.

CRS: Bush-Francis Catatonia Rating Scale; F: female; M: male

Baclofen doses of more than 200 mg are associated with coma, delirium, and seizures. Toxicologic studies revealed a clinical spectrum of baclofen overdose involving encephalopathy, respiratory depression, muscular hypotonia, and generalized hyporeflexia. Interestingly, this was mainly observed in individuals with renal impairment. Nahar *et al*. reported a patient with catatonic features in addition to psychosis following a baclofen overdose [2]. Psychosis with catatonic features was already described with other muscle relaxants. In 1983, Beeber and Manring probably depicted the first case of catatonia secondary to a muscle relaxant [8]. They reported a 38-year-old female who received cyclobenzaprine for back pain. She developed manic psychosis and some catatonic symptoms (posturing and echolalia). Noteworthy, the patient had a previous significant psychiatric medical history, including manic episodes.

**Figure 1:**
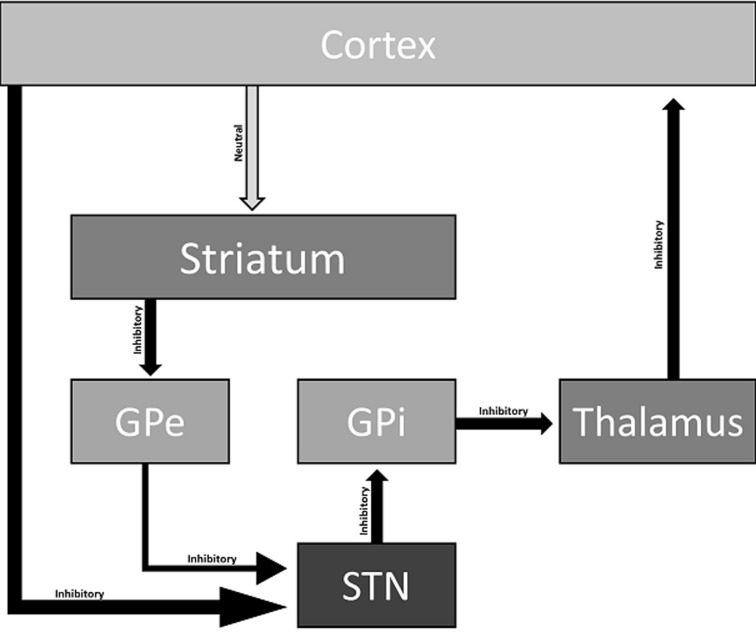
possible pathophysiological mechanism for baclofen-induced catatonia

A possible explanation for baclofen-induced catatonia is the interaction of baclofen in GABA receptors in the basal ganglia (Figure 1). The motor control involves GABAergic and glutamatergic neurons in the cortico-striato-pallido-thalamo-cortical loop. In this way, the increased GABAergic activity can lead to direct or indirect inhibition of dopamine release. This modulation of firing dopamine neurons could contribute to developing psychotic and motor symptoms characteristic of catatonia [9]. Moreover, mania-like symptoms can be explained by an increased noradrenergic turnover by altering the firing rate of GABAergic neurons [10]. Another hypothesis is serotoninergic disinhibition, which can partially explain some mania-related symptoms. It is believed that presynaptic GABA-B receptors interaction in the dorsal raphe nucleus can increase serotonin release [3].

A more recent hypothesis for explaining catatonia involves a disbalance between GABA receptors. In rat models, it was observed that hyperactivity at the GABA-B receptor and hypoactivity at the GABA-A receptor could cause catatonia symptoms [9]. In this context, baclofen may lead to increased selective activity of GABA-B receptors in susceptible individuals [1]. It is worth mentioning that this pathway can explain hypokinetic catatonia but does not explain exciting types of catatonia. To be more specific, borderline forms like periodic catatonia and delirious mania are not supported by this pathophysiological mechanism [9].

## Conclusion

Catatonia secondary to baclofen was rarely reported in the literature. Most of the affected patients have a previous medical history of psychotic symptoms and catatonia. The mechanism for explaining baclofen-induced catatonia probably involves increased GABAergic neurotransmission or a disbalance between different GABA receptors. Clinicians should be aware of this possible side effect to prompt diagnosis and short-term recovery.
